# Toxicity, quality of life, and PSA control after 50 Gy stereotactic body radiation therapy to the dominant intraprostatic nodule with the use of a rectal spacer: results of a phase I/II study

**DOI:** 10.1259/bjr.20220803

**Published:** 2023-03-03

**Authors:** Minna Cloitre, Massimo Valerio, Ange Mampuya, Arnas Rakauskas, Dominik Berthold, Thomas Tawadros, Jean-Yves Meuwly, Leonie Heym, Frederic Duclos, Véronique Vallet, Michele Zeverino, Patrice Jichlinski, John Prior, Beat Roth, Jean Bourhis, Fernanda G Herrera

**Affiliations:** 1 Department of Oncology, Radiation Oncology Service, Centre Hospitalier Universitaire Vaudois, Lausanne, Switzerland; 2 Department of Surgery, Urology Service, Centre Hospitalier Universitaire Vaudois, Lausanne, Switzerland; 3 Department of Oncology, Medical Oncology Service, Centre Hospitalier Universitaire Vaudois, Lausanne, Switzerland; 4 Department of Radiology, Centre Hospitalier Universitaire Vaudois, Lausanne, Switzerland; 5 Department of Radiology, Nuclear Medicine Service, Centre Hospitalier Universitaire Vaudois, Lausanne, Switzerland

## Abstract

**Objectives:**

We conducted a phase I/II prospective trial to determine whether stereotactic dose escalation to the dominant intra-prostatic nodule (DIN) up to 50 Gy incorporating a rectal balloon spacer is safe, does not affect patient quality of life, and preserves local control in patients with intermediate-high risk PCa.

**Methods:**

Eligible patients included males with stage ≤T3b localized disease, a prostate-specific antigen (PSA) level ≤50 , International Prostate Symptom Score (IPSS) ≤14, and a gland volume ≤70 cm^3^. Patients underwent perirectal spacer placement, followed by a planning MRI and were subsequently treated with SBRT doses of 36.25 Gy in five fractions to the whole prostate while simultaneously escalating doses to the magnetic resonance image visible DIN up to 50 Gy. Primary endpoint: safety. Secondary endpoints: biochemical control, quality of life (QofL), and dosimetry outcome.

**Results:**

Nine patients were treated in the Phase I part of the study. Dose limiting toxicities (DLTs) were not observed. Further characterization of tolerability and efficacy was conducted in the subsequent 24 patients irradiated at the recommended Phase II dose (50 Gy, RP2D). At a median follow-up of 61 months, biochemical control is 69%. Grade 1 and 2 acute GU and GI toxicity was 57.5 and 15%, and 24.2 and 6.1%, respectively. Grade 1 and 2 late GU and GI toxicity was 66.6 and 12.1%, and 15.1 and 3%, respectively. No Grade 3 or higher toxicity was reported. QofL data confirmed physician’s reported side effects. Dosimetry analysis showed adherence to the doses prescribed in the protocol.

**Conclusions:**

SBRT of the whole prostate with 36.25 Gy in 5 fractions and dose escalation to 50 Gy to the DIN, when combined with a peri-rectal balloon spacer, was tolerable and established the RP2D. QofL analysis showed minimal negative impact in GU, GI, and sexual domains.

**Advances in knowledge:**

Extreme hypofractionated prostate radiation therapy with focal dose escalation to the DIN is well tolerated with efficacy comparable to normal fractionated radiation therapy.

## Introduction

Prostate cancer (PCa) is the third most common cancer in the world, with 1.4 million new cases identified each year, and around 90% of them have a localized tumor.^1^

For all risk scenarios of localized PCa, radiotherapy (RT) is a well-established, potentially curative therapeutic option. Combining androgen deprivation therapy (ADT) with RT has been proven to increase overall survival in patients with intermediate- and high-risk PCa.^2–4^ Several studies have shown that dose-escalated external beam RT (EBRT) has better biochemical control than standard-dose EBRT,^5,6^ and dose-escalation with brachytherapy boost may be even better than dose-escalated EBRT.^7–9^

Since dose-escalated RT normally necessitates an 8-week total treatment duration and brachytherapy is an invasive technique with limited availability at many RT facilities, shorter and more convenient treatment regimens are needed. Stereotactic body radiation therapy (SBRT) is a highly conformal RT method that enables for ultra-hypofractionation, which significantly reduces overall treatment time. SBRT can be safely delivered to the whole prostate gland and seminal vesicles employing PTV prescription doses of 36.25 Gy or 40 Gy administered in five fractions (7.25 Gy or 8 Gy per fraction) with excellent results in patients with low- and intermediate-risk PCa.^10–14^ There is, however, paucity of clinical evidence on SBRT dose escalation in patients with high-risk PCa. For instance, in the HYPO-RT-PC trial,^12^ the 5-year disease control rate following ultra-hypofractionated RT was 84%, with 1054 (89%) patients being intermediate risk and 126 (11%) being high risk; findings for the high-risk subgroup have not yet been published.

New imaging techniques, such as multiparametric magnetic resonance imaging (mpMRI), have significantly improved the sensitivity and specificity for identifying and characterizing high-risk PCa foci.^15^ For example, mpMRI can detect the dominant intraprostatic nodule (DIN), which is the largest nodule with the most aggressive biological behaviour, and studies of patterns-of-failure after conventional EBRT have revealed that the DIN was the main site of tumor recurrence in more than 90% of the patients.^16,17^ As a result, the DIN is an ideal target for heterogeneous dose escalation using SBRT, particularly given that previous landmark trials^18,19^ of 50 Gy to the entire prostate resulted in an increase in severe late toxicity, with 5.5% of the patients requiring temporary diverting colostomy at 9-month follow-up and a significant correlation between high-grade toxicities and volume of rectal wall receiving 50 Gy (V50>3 cm^3^).^19^ We report here the findings of a Phase I/II trial in which we used SBRT to irradiate the whole prostate gland with tumoricidal doses of 36.25 Gy in five fractions while simultaneously increasing the radiation dose to the DINs up to 50 Gy. To achieve this, the protocol mandated the use of a rectal balloon spacer to maximize rectal protection. Importantly, 97% of our patients harboured intermediate and high-risk PCa and only one of them received ADT.

## Patients and methods

This trial is a Phase I/II dose escalation approved by the Ethics Committee of Canton Vaud (ClinicalTrials.gov ID: NCT02254746). All patients provided written informed consent. Eligible patients, enrolled between October 2014 and April 2017, were those with newly diagnosed and previously untreated PCa, low, intermediate- and high-risk disease according to the D'Amico risk classification,^20^ and stage T2 to T3 adenocarcinoma of the prostate, N0, M0. All patients had to have at least one visible nodule at mpMRI. The serum PSA level was required to be <50 µg/l, and the International Prostate Symptom Score (IPSS)≤15 (α blockers allowed). Patients were excluded if they had a pre-SBRT prostate volume on MRI greater than 70 cm^3^ or a tumor located at less than 3 mm from the urethra when measured at the mpMRI. They were also excluded if they had evidence of inflammatory colitis or previous RT in the pelvis. Concomitant or adjuvant ADT was allowed, but neoadjuvant ADT was an exclusion criterion.

The primary endpoint of the study was to assess acute (up to 90 days after the first RT fraction) urinary and rectal toxicity. Secondary endpoints were biochemical control, quality of life (QofL), and dosimetry outcomes.

## Radiotherapy planning and delivery

A biodegradable spacer (BioProtect Balloon^™^ Implant system, BioProtect Ltd., Tzur Igal, Israel) was trans-perineally implanted between the prostate and the rectum under transrectal ultrasound guidance with the patient under sedative anesthetics. During the same surgery, four gold anchor fiducial markers (Gold Anchor, Naslund AB, Sweden) were inserted in the prostate with at least 2 cm of space between them to meet the fiducial spacing threshold of at least 1 cm on orthogonal imaging to assure precise rotational corrections. To meet the collinearity criteria, all angles formed by at least three fiducials have to be greater than 15°. The planning MRI and planning computed tomography (CT) scans were performed between 1 and 7 days following fiducial and balloon insertion. To avoid anatomic changes in the rectum that could interfere with image fusion, the planning MRI was immediately followed by a planning CT scan. To favor an accurate fusion both, planning MRI and planning CT, were performed with the same slide thickness of 1 mm.^21^ The planning *T*_2_-weighted MRI image sets after rectal spacer/fiducial markers insertion were rigidly fused to the planning-CT images (fiducials-based registration), and no catheter was used to visualize the urethra. This was done to ensure accuracy on contouring and appropriate visualization of the DIN and organs at risk (OARs).

During the planning scans and treatment, precautions were taken to reduce prostate motion. Patients were instructed to maintain a low-fiber diet beginning 5 days before the planning scans and continuing until the completion of treatment to decrease intestinal gas. They were required to take a moderate laxative 48 h before to the planned MRI and CT scan. If necessary, enemas were performed 1 h before the acquisition of the planning scans and before each treatment session as needed to reduce rectal volume. Patients were also encouraged to drink 200 ml of water 1 h before the scans, after voiding completely. The lead investigators of the trial (FH & JB) draw and/or supervised the anatomical contours of the prostate, DIN, seminal vesicles, and OARs, which were then reviewed by a panel of board-certified radiation oncologists. The DIN and the urethra were identified as the region of interest in the MRI by a radiologist (J-YM). To generate the planned target volume, the prostate was uniformly expanded by 3 mm (PTVp).

The DIN was contoured as the gross tumor volume (GTV) and expanded by 3 mm to create a PTV_DIN_ (no clinical target volume was used around the GTV). The prescribed dose to the PTVp was 36.25 Gy in five fractions (7.25 Gy per fraction). The prescribed dose to the PTV_DIN_ was 45, 47.5, and 50 Gy in five fractions, corresponding to the 80% isodose line; therefore, the maximum dose point corresponded to 56.25, 59.38, and 62.5 Gy, respectively. To allow gradients for DIN boosting and to maximize PTV_DIN_ doses, there were no limits on dose heterogeneity. At least 95% of the volume of interest (PTV_DIN_ and PTVp) had to be covered by >95% of the prescription dose. A minimum of 2 days and a maximum of 6 days had to separate each treatment fraction. No more than two fractions would be delivered per week. The overall treatment time had to be no more than 26 days.

Dose-volume histogram goals and details of RT planning were published elsewhere (HERE PLEASE INCLUDE THE FOLLOWING CITATION doi: 10.1016/j.ijrobp.2018.09.023 Herrera F.G et al THIS CAN BE INCLUDED MANUALLY AS NEW REF 47. Briefly, Dose-volume histogram goals for the rectum were maximum dose to to 0.1 cm^3^ <41 Gy, and V25 <20 (*i.e.,* the volume receiving 25 Gy <20 cm^3^). The bladder dose-volume histogram was limited to no more than 0.1 cm^3^ to receive less than 45 Gy, and the bladder median dose was not to exceed 20 Gy. The urethra dose was limited to no more than 1 cm^3^ of urethra receiving more than 39 Gy and 0.1 cm^3^ not to exceed 41 Gy.

Whenever possible, patients had to be treated with CyberKnife. However, Tomotherapy (Accuray Inc, Sunnyvale, CA) or VMAT (Elekta Synergy Stockholm, Sweden) were allowed in case of impossibilities to treat with CyberKnife (*e.i*. machine break down or temporary unavailable). Patients were treated with an energy of 6 MV. Orthogonal X-ray imaging was used for image guidance based on fiducial markers position. In patients treated with Cyberknife, the shift of X-ray images from the planning CT scan was monitored in real time during each fraction. In patients receiving Tomotherapy or VMAT, the RT session was interrupted every 10 min to allow for re-scanning and verification of the position of the prostate and fiducial markers.

To improve patient comfort, all patients were treated in the supine treatment position with a knee cushion.

## Treatment schema and statistical analysis

The radiation dose to the whole prostate was 36.25 Gy in 5 fractions of 7.25 Gy. For the Phase I part of the study, dose escalation to the DIN was performed using the traditional 3 + 3 design.^22^ Dose limiting toxicities (DLTs) were defined as Grade 3 or higher gastrointestinal (GI) or genito-urinary (GU) toxicity that appeared from the first fraction of RT and up to 90 days after completing treatment using the National Cancer Institute Common Terminology Criteria for Adverse Events (NCI-CTCAE, version 4) up to 90 days after the first radiation fraction. Patients were treated with a starting dose to the DIN of 9 Gy per fraction up to 45 Gy. If no DLT was observed in the first cohort, an additional three patients were entered at the next dose level (47.5 Gy in 5 fractions of 9.5 Gy), with dose escalation continuing until DLT was observed or if the maximum tolerated dose (MTD, 50 Gy in 5 fractions of 10 Gy) was reached in the absence of a DLT. If one of the three patients experienced a DLT at a particular dose level, an additional three patients were planned to be included at that level. If two or more patients experienced a DLT at a given dose level, a lower dose level would have been explored to define the MTD. The three patients included in a cohort could be enrolled simultaneously or sequentially without any waiting period among them. However, dose escalation in the Phase I was not allowed until the last patient included in a specific cohort completed a minimum follow-up period of 90 days without experiencing DLT. After the Phase I part of the trial was completed, an interim analysis was performed, and the trial was examined by an Independent Data Safety Monitoring Board (IDSMB) in order to authorize its continuation to Phase II. Patients treated in Phase I at the MTD or DLT would be included in the Phase II analysis.

A Simon optimal two-stage design^23^ was used to calculate sample size for the Phase II part of the study, using acute GU and GI toxicity of equal or more than Grade 2, occurring during treatment and up to 90 days after completion of SBRT. We consider an acute Grade 2 or more toxicity rate of 10% as acceptable for the treatment to be promising, while a Grade 2 or more toxicity rate of 70% would be unacceptable. With a probability of 5% of accepting the treatment as acceptable if true toxicity-free rate is equal or less than 70% (α) and a probability of 20% of rejecting the treatment if the true toxicity-free rate is equal or more than 90% (β). The first six patients treated at the recommended Phase II dose (RP2D) would be subjected to an interim safety analysis performed by the IDSMB. If two or more patients out of six treated would develop Grade 2 or more GU or GI toxicity, the trial would be stopped, otherwise 21 additional patients would be accrued into a second stage. After a total Phase II sample size of 27 patients treated the treatment would be promising for further study if five or fewer patients would have developed GU or GI toxicity of Grade 2 or more within the first three months of SBRT (Supplementary Figure 1).

Supplementary Figure 1.

Secondary endpoints were late toxicity (occurring>90 days from first fraction), PSA kinetics, and patient-reported outcome. European Organization for Research and Treatment of Cancer QoL Form PR25 (prostate module) together with the IPSS score were collected at baseline and 1, 3, and 6, months after treatment. After 6 months QofL evaluation was not mandated by the trial but collected whenever possible. Health-related QoL outcomes were scored using the European Organization for Research and Treatment of Cancer guidelines^24^ into values ranging from 0 to 100. A difference of 10 points or more was considered clinically relevant.^25^ Patients were followed up by having PSA measurements, and a physical examination performed every 3 months for the first 2 years, and every 6 months thereafter. The nadir+2 failure definition was used for biochemical control.^26^ Upon confirmation of biochemical recurrence, patients were staged using Gallium-68 prostate-specific membrane antigen positron emission tomography-computed tomography (Ga-68 PSMA PET/CT) and whenever necessary prostate mpMRI. Analyses were carried out in Prism 7 (GraphPad Software, Inc, La Jolla, CA).

## Results

### Patient characteristics

Available data, toxicity, patient reported outcome and PSA control were obtained for 33 patients included in the trial, of whom 9 were included in Phase I and 24 in Phase II. Patient median age was 71 (range: 55–83). 94% of the patients were stage T2a-T3b. Median PSA was 12.83 µg/l (range: 2.7–40.4 µg/l). 54.6% were high risk disease, 42.4% were intermediate risk, and 3% low risk. Median IPSS was 5 (range 0–24) and median prostate volume was 40 ml (range: 12.4–67 ml). The median number of DIN was 1 (range: 1–3) and their median volume was 1.96 ml (range: 0.37–4.20). With the aid of a rectal balloon spacer the median distance between the DIN and the rectum was 12.2 mm (range: 4–22.9 mm). The median distance between the DIN and the urethra was 7.6 mm (range: 3–16.9 mm).

The median RT treatment duration was 19 days (range: 11–26 days). All patients were able to complete their treatments. Despite appropriate counseling 97% of the patients refused ADT; ADT was only administered to a patient with intermediate-risk disease for 6 months. Patient’s and tumor characteristics are described in Table 1.

**Table 1.** Tumor and Treatment Characteristics

Characteristics	N	%
**T stage**		
T1c	2	6.1
T2a	10	30.3
T2b	9	27.3
T2c	8	24.2
T3a	3	9.1
T3b	1	3
**Gleason Score**		
3 + 3	3	9.1
3+4	17	51.5
4+3	5	15.2
4+4	4	12.1
3+5	1	3
4+5	3	9.1
Low risk (GS < 6 and PSA < 10 and T1c/T2a)	1	3
Intermediaterisk(GS = 7 or PSA > 10 or ≤cT2a)	14	42.4
High risk (GS ≥ 8 or ≥cT2c or PSA > 20)	18	54.6
**Hormonal therapy**		
Yes	1	3
No	32	97
**Radiotherapy delivery**		
CyberKnife	27	81.8
Tomotherapy	3	9.1
Synergy	3	9.1

### Side effects

In the Phase I part of the study, no DLT was identified within 90 days of therapy initiation; hence, dose escalation continued through all intended dose levels. As a result, three patients were enrolled in each dose level (*N* = 9) and 24 more patients were enrolled at the RP2D (50 Gy). Within the first 3 months of SBRT completion, 15 and 6.1% of the patients showed treatment-related Grade 2 GU and GI toxicity, respectively.

The number of patients experiencing GI and GU toxicity by grade and time is shown in Table 2. Supplementary Table 1 shows the most common reported acute GU and GI adverse events. Only Grade 1 and 2 GI and GU toxicity was observed within 90 days. No Grade 3 toxicity occurred over the complete course of follow-up in any of the recruited patients. No complications or side-effects were observed because of the placement of the rectal balloon spacer. Late GU and GI Grade 1 and 2 toxicity occurred in 66.6%, 12.1%, 15.1%, and 3% of patients, respectively (Supplementary Table 2).

Supplementary Table 1.

**Table 2.** Genitourinary and gastrointestinal toxicity according to grade and time.

Grade	GU toxicity				GI toxicity			
	≤90 days		>90 days		≤90 days		>90 days	
	*N*	%	*N*	%	*N*	%	*N*	%
1	**19**	57.5	**22**	66.6	**8**	24.2	**5**	15.1
2	**5**	15	**4**	12.1	**2**	6.1	**1**	3

GI, gastrointestinal toxicity; GU, genitourinary toxicity.

*N*: number of patients having developed grade 1 or 2 toxicity (total number of patients = 33)

Toxicity graded according to Common Terminology Criteria of Adverse Events, version 4.

No grade 3 or more toxicity was reported.

Time period: from day 1 of stereotactic radiation therapy and up to 90 days after the first fraction or more than 90 days after the first radiation fraction.

### Efficacy

**Figure 1.**
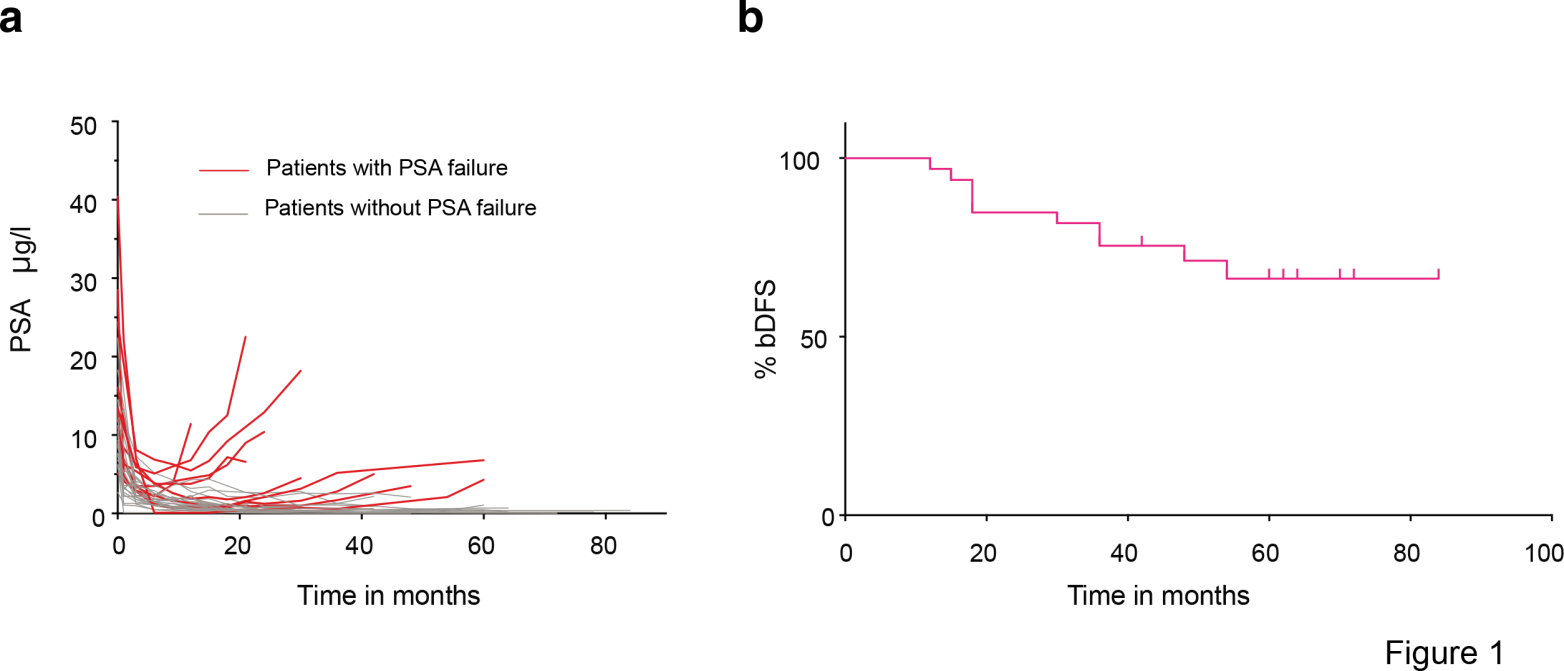
(**A**) Prostate-specific antigen (PSA) kinetics in micrograms per Liter (µg/l), PSA failure-positive patients in red (*n* = 10), PSA failure-negative patients in grey (*n* = 23. (**B**) Biochemical disease-free survival (bDFS) in 33 patients included in the study.

At a median follow-up time of 61 months (range 48 to 110 months), 10 out of 33 patients had a PSA recurrence (30.3%), (Figure 1A **and** Figure 1B). Upon recurrence, all patients underwent a Ga-68 PSMA PET/CT in order to determine the site of recurrence. The relapse pattern in the 10 biochemically relapsed patients was as follows:

Two patients had distant metastases only; three patients had lymph node metastasis only; one patient had lymph node metastasis with concomitant intraprostatic in-field recurrence; two patients had in-field prostate recurrence only; and two patients had biochemical failure without evidence of metabolically active disease. Among the three patients with a local intraprostatic recurrence, one of them recurred in the area of the SIB-DIN which received 50 Gy while the other two patients recurred outside the SIB-DIN in the area that received 36.25 Gy and had no MRI recognizable nodule prior to treatment.

Among the 10 patients with recurrence, 40% had an initial T3a/b disease and/or Gleason score≥8. Three patients died during study follow-up: one from metastatic PCa, one from COVID-19, and one from head and neck cancer. Deaths occurred after patients completed the first 3 months toxicity period evaluation (primary endpoint).

### Patient’s reported outcome

**Figure 2.**
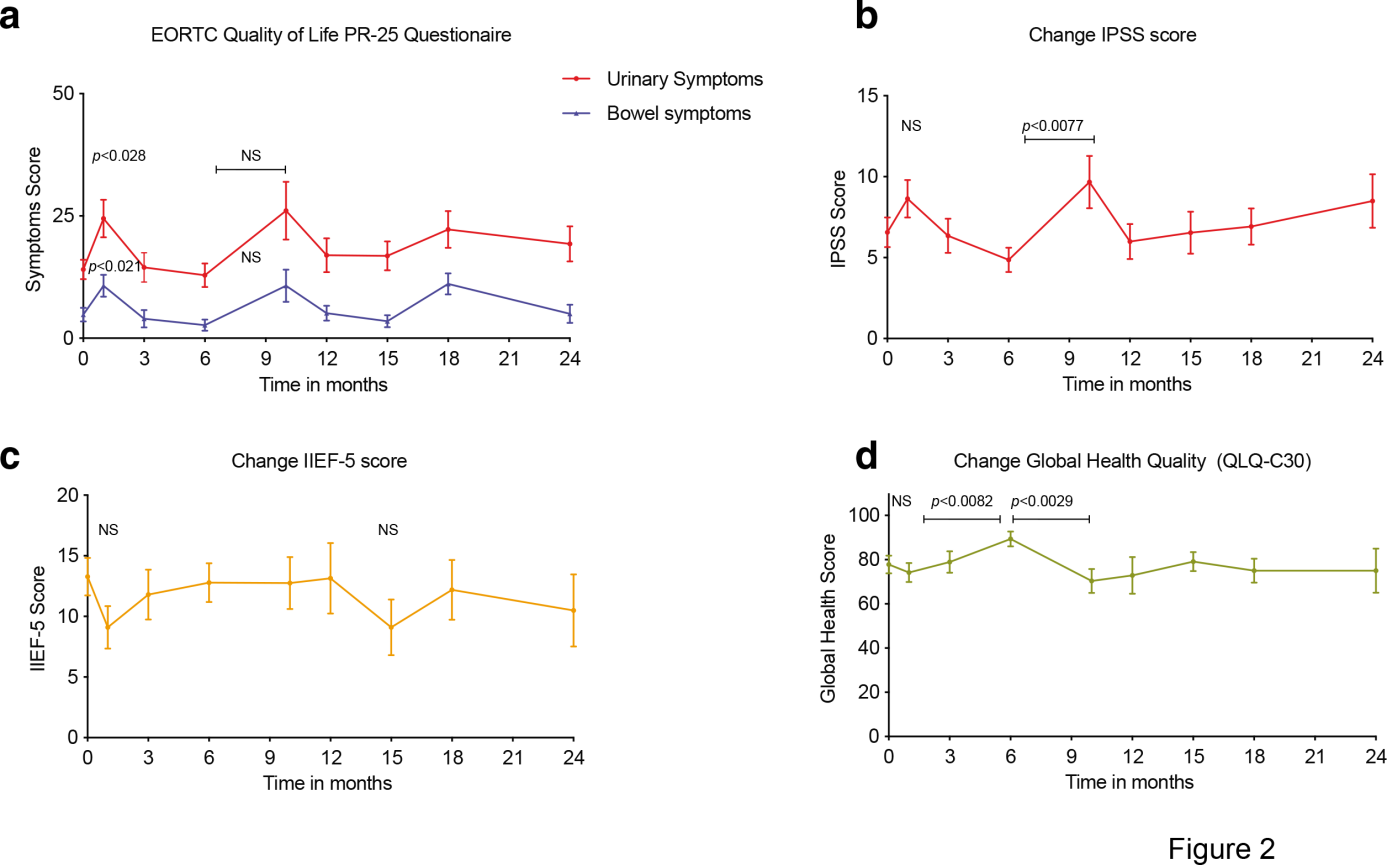
Patient-reported quality of life (with standard error of the mean) over time: (**A**) urinary (red line) and bowel (blue line) symptoms using the PR-25-EORTC questionnaire. (**B**) Urinary quality of life using the International Prostate Symptom Scoring (IPSS) system. (C) Erectile dysfunction using the International Index of Erectile Function (IIEF-5) questionnaire. (D) General health-related quality of life evaluation using the cancer-specific EORTC QLQ-C30 questionnaire.

The QofL GI-PR25 scores increased from 4.83 at baseline to 10.71 points one month after therapy (*p* < 0.021), before returning to normal values three months later and further increasing at 10 and 18 months before stabilizing. In terms of GU QofL scores, they increased from 14.06 points at baseline to 24.7 points one month after therapy (*p* < 0.028), returning to baseline values at month 3 and increasing further at months 10 and 18 while stabilizing thereafter (Figure 2A). The IPSS score, which increased from 6.56 points at baseline to 8 points one month after therapy (NS *p*-value) followed the same patterns as the GU PR-25 score with a subsequent increase from 4.85 point at 6 months to 9.66 points at 10 months (*p* < 0.0077) (Figure 2B). IIEF-5 functional levels fell at 1 month and 15 months after therapy, although this decline was not statistically significant (Figure 2C). Notably, the patient’s global health status decreased slightly in the month following treatment before increasing at 3 months (*p* < 0.0082), declining at 9 months (*p* < 0.0029), and stabilizing thereafter (Figure 2D).

### Dosimetry analysis

Table 3 and Table 4 show the planned versus the delivered doses to the organs-at-risk and the targeted volumes, compliance with dosimetry protocol requirements was achieved in 100% of the patients. Since there were no changes to the rectal spacer during treatment, SBRT treatment re-planning was not necessary

**Table 3.** Dosimetry analysis for targeted volumes

Target volume	Median dose Gy (ranges)	Minimum dose Gy (ranges)	Maximum dose Gy (ranges)
PTV_DIN_ Phase Ia	50 (47 – 55)	41 (34 – 44)	56.5 (range 50–62)
PTV_DIN_ Phase Ib	54 (47 – 57)	45 (32 – 50)	61 (50 – 63)
PTV_p_	53 (46 – 56)	44 (31 – 49)	60 (50 – 62)

NA, not applicable; PTV_DIN_, planning target volume of the dominant intra-prostatic nodule ; PTV_p_, planning target volume of the prostate.

**Table 4.** Dosimetry analysis for the organs at risk

Organ at risk	Dose planned per protocol	Median Delivered (range)
Rectum	0.1 cm^3^ < 41 Gy V25 Gy < 20 cm^3^	0.1 cm^3^< 32.8 Gy (25.2–41 Gy) V25 Gy < 1.6 cm^3^ (0.1–20 cm^3^)
Bladder	0.1 cm^3^< 45 Gy Median dose < 20 Gy	39.9 Gy (36–44 Gy) 14.5 (4.76–21.6 Gy)
Urethra	0.1cm^3^ < 41 Gy 1 cm^3^ < 39 Gy	38.8 Gy (35–41 Gy) 37.2 Gy (33.4–39 Gy)

## Discussion

Several prospective trials, notably the HYPO-RT-PC Phase 3 randomized trial (ISRCTN 45905321) and early results on toxicity and QofL from the PACE-B study (NCT01584258), have established SBRT as a standard-of-care treatment option for patients with localized PCa. Previous pivotal trials of dose escalation to the whole prostate with 45 Gy and 47.5 Gy have shown no evidence of severe toxicity.^18,19,27,28^ However, the DLT threshold has been established at 50 Gy which resulted in an increase in severe GU and GI toxicity.^18,19^ Meanwhile, several studies justify the interest of increasing prostate SBRT doses. For instance, the Jackson et al meta-analysis confirmed^14^ the advantage of SBRT dose escalation, demonstrating that increasing SBRT dose was associated with improved biochemical control (*p* = 0.018). Similarly, other prospective studies^27^ have demonstrated that increasing the dose of SBRT leads to lower rate of positive posttreatment biopsies. Furthermore, results from the FLAME trial,^29^ which used a focal EBRT microboost to 95 Gy and demonstrated improvements in biochemical control with no excess toxicity, suggest that intensive dose escalation to the whole-gland could be replaced by a focal-directed dose escalation to the DIN, which is the area at the highest risk of recurrence. The fundamental obstacles of dose escalation to the DIN continue to be the safety of SBRT heterogeneous dose distribution, particularly given the difficulty in curtailing hotspots near healthy organs (urethra, bladder, and rectum), as well as the uncertainty associated with organ movement. We, therefore, performed a Phase I/II study in order to study dose escalation up to 50 Gy to the DIN and show the safe delivery of this escalated doses.

Concerning genitourinary toxicity, we found Grade 1 and 2 acute toxicity in 57.5% and 15% of the patients, respectively. Reported rates of acute Grade 1 or 2 GU toxicity in previous SBRT trials that limited the dose to 35–36.25 Gy were between 28 and 78%.^30–35^ In the HYPO-RT-PC^12^ randomized Phase III trial which delivered 42.7 Gy in seven fractions, acute Grade 2 or worse GU toxicity occurred in 28% of the patients in the ultra-hypofractionated group *vs* 23% in the conventional fractionated arm (*p* = 0·057). Studies administering 50 Gy with CyberKnife reported Grade 1–2 acute GU toxicities of 50 to 60%. (Here insert please reference 36 Kotecha ^36,37^ Boike et al reported Grade 1–2 acute GU toxicity of 60% when delivering 50 Gy to the whole prostate.^18^

In our trial, Grade 1 and 2 late GU toxicities was 66.6 and 12.1%, respectively. The landmark HYPO-RT-PC randomized trial^12^ found a 5% incidence of Grade 2 or worse urinary toxicity after a median 5 years follow-up in both the ultra-hypofractionation and conventional fractionation groups. Folkert et al^37^ recently presented the findings of a Phase II study which aimed to escalate the dose to 45 Gy in 5 fractions of 9 Gy to the whole prostate. After a median follow-up of 48 months, 33.3% of patients experienced Grade 2 late GU toxicities. Similarly, a series of 8 consecutive patients treated at Royal Marsden with CyberKnife using 36.25 Gy to the whole prostate with SIB to the DIN 47.5 Gy reported 12.5% late GU toxicities at a median follow-up of 56 months. Notably, a patient in that study had Grade 3 cystitis (hematuria).^38^ Thus, our late toxicity rate is slightly higher than the ones reported in previous trial consistent with the higher doses delivered. What it is of most importance is that our patients did not developed Grade 3 or more acute or late GU toxicity.

Our study found that acute Grade 1 and 2 GI toxicity occurred in 24.2 and 6.1% of patients, respectively. Previous SBRT trials that irradiated the whole prostate gland with a dose of 35 to 40 Gy found acute Grade 1 to 2 GI toxicity rates ranging from 16 to 82%.^30–35^ Boike et al^18^ reported 54% acute Grade 1–2 GI toxicity when irradiating the whole prostate with 50 Gy which subsequently translated in severe GI toxicity.^18^ Inspired by the Boike et al trial our study mandated the use of a rectal spacer which we believe significantly contributed to the reduced rates of acute and late GI toxicities, for example we reported only 3% late G2 GI toxicity which is quite unusual when escalating RT doses to the prostate. Folkert et al, reported late Grade 2 or more GI toxicity in 14.3% of patients treated with five 9 Gy fractions using a rectal spacer.^37^ In that study, the median spacing between the prostate and the anterior rectal wall that was created by the spacer was 11.3 mm (9–13.1 mm), which was comparable to the one observed in our study (12.2 mm, range: 4–22.9 mm^37^), therefore it is possible that the use of precise irradiation technique and a rectal spacer were responsible for the reduced GI toxicity observed in our study. Rectal spacers have been demonstrated to improve dosimetric and toxicity outcomes, and retrospective SBRT studies have investigated their use.^39–41^ In a study that included 50 patients, Hwang et al^42^ described results using a hydrogel rectal spacer using 36.25 Gy in five fractions. They found minimal acute Grade 2 toxicity (4%) and no late GI toxicity of Grade 2 or more after a median follow-up of 20 months. Thus, we believe that the relatively low rates of GI toxicity reported in our trial are due, in part, to the use of a mandatory rectal spacer, which allowed us to keep our dosimetric constraints below the rectal dose limits previously reported by Kim et al as predictors of rectal toxicity (>3 cm^3^ of the anterior rectal wall exposed to 50 Gy or >35% of the rectal wall circumference exposed to 39 Gy^19^).

In our study, patient-reported outcomes and clinician-reported assessments were performed with the aim of being complementary and to more fully document the burden of toxicities and subjective symptoms, such as sexual dysfunction. We observed minimal declines in PR25 urinary and bowel symptoms score as well as IPSS score.

The HYPO-RT-PC trial recently reported the results of QofL comparisons between ultra-hypofractionated and conventional fractionated-RT, demonstrating that ultra-hypofractionation was as well tolerated as conventional fractionation up to 6 years after treatment completion.^43^ Jackson et al^14^ obtained QofL data from 25 SBRT studies (*n* = 3973). Expanded Prostate Cancer Index Composite urine and bowel scores declined by 10 points during treatment and reverted to baseline by 2 years post-treatment.^14^ Early findings from the randomized non-inferiority Phase III trial PACE-B,^11^ which compared SBRT to moderately hypofractionated RT or conventionally fractionated RT, revealed no differences in QofL between the three treatments, with EPIC index reductions ranging from 5 to 8 points for the GU and GI domains, respectively.^11^

Our study QofL analysis truly reflected physicians reported outcome and are in line with that previously reported in the literature.

It’s tempting to look at clinical outcomes in our trial, especially since we included a significant proportion of high-risk patients and ADT was omitted in all of them. However, since our study was not powered to assess clinical effectiveness, any interpretation of the biochemical relapse-free survival outcome should be done with caution. We observed PSA relapses in 30% of the patients at a median of 61 months. Most of the recurrences observed were in patients with high-risk disease. This failure rate is comparable to that reported by Lee et al,^44^ who recruited 45 patients in a Phase II trial, 28.8% of whom had high risk disease (T3 or Gleason 8–10 or PSA>20 ng/ml), and reported an 89.7% biochemical relapse free survival after a median follow-up of 63 months. Although ADT is undoubtedly standard-of-care in this patient population, our patients refused it, probably in the hopes of getting prolonged remissions with SBRT. According to a recent SEER National Database retrospective study,^45^ SBRT use has increased dramatically for males with high-risk PCa in the US, and this increase has been mostly driven by increased utilization among males who are not undergoing concomitant ADT. It is possible that the patients in our study refused ADT in the assumption that higher radiation doses to the prostate would give equivalent disease control with better QoL, especially sexual QofL.

Several limitations of our study are acknowledged. The sample size is insufficient to draw firm conclusions. The follow-up period is still insufficient to assess the biochemical or survival outcome. Another limitation is that we decided to exclude patients with IPSS scores greater than 14, tumors near the urethra, or prostate sizes greater than 70 cm3, thus our findings cannot be extended to all patients with intermediate-high risk PCa.

## Conclusions

Dose escalation to the DIN of up to 50 Gy was feasible and safe. Favorable rates of urinary and rectal toxicities were attained using 50 Gy SBRT, which mirrored patient-reported outcome data. There was no toxicity of Grade 3 or higher observed. Preliminary tumor control rates in patients with intermediate-high risk disease are encouraging, but no firm conclusions can be drawn due to the still short follow-up, the small number of patients as well as the lack of ADT administration. The excellent safety outcome of our study was most likely due to the substantial efforts made to protect healthy tissues from high doses of radiation, as well as the highly selected patient population recruited in this study.
