# Supplementary figures and images for: Toxicity, quality of life, and PSA control after 50 Gy stereotactic body radiation therapy to the dominant intraprostatic nodule with the use of a rectal spacer: results of a phase I/II study
Source: Br J Radiol. 2023 Mar 3;96(1145):20220803. doi: 10.1259/bjr.20220803 (PMC10161910; doi:10.1259/bjr.20220803)

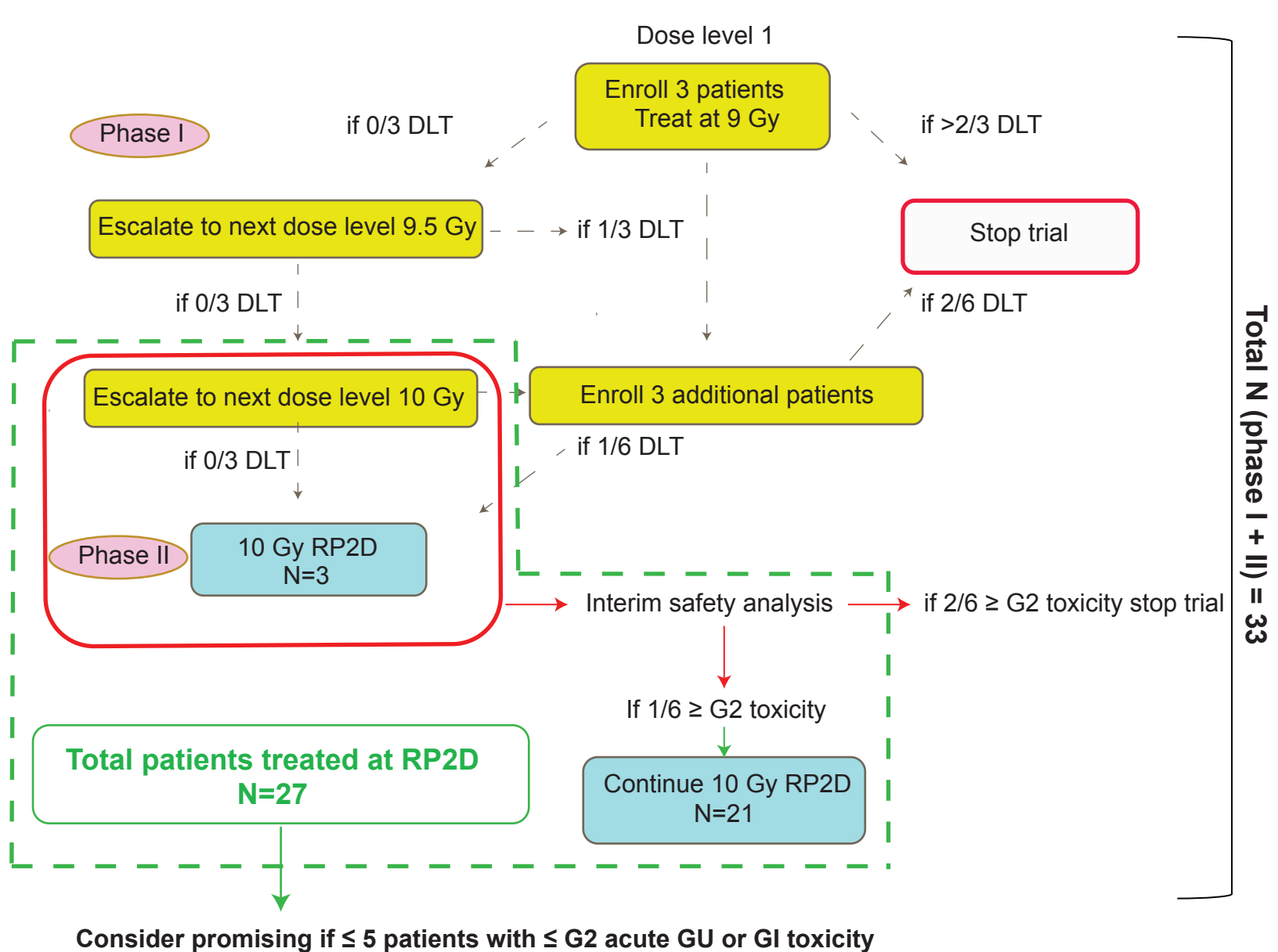

Supplementary Figure 1

Supplement: Supplementary Figure 1. [file bjr.20220803.suppl-01.pdf]
