# Supplementary material for: Toxicity, quality of life, and PSA control after 50 Gy stereotactic body radiation therapy to the dominant intraprostatic nodule with the use of a rectal spacer: results of a phase I/II study
Source: Br J Radiol. 2023 Mar 3;96(1145):20220803. doi: 10.1259/bjr.20220803 (PMC10161910; doi:10.1259/bjr.20220803)
Supplement: Supplementary Table 1. [file bjr.20220803.suppl-02.docx]

**Supplementary Table 1**. Acute toxicity profile (a patient may be counted more than once).

| **Acute toxicity profile G1-2** | **Phase I**  **9 Gy**  **N=3** | **Phase I**  **9.5 Gy**  **N=3** | **Phase I/II**  **10 Gy**  **N=27** |
| --- | --- | --- | --- |
| **Gastrointestinal** |  |  |  |
| Diarrhea | 1 | 2 | 3 |
| Constipation | 0 | 1 | 0 |
| Anal mucositis | 0 | 1 | 0 |
| Anal pain | 0 | 0 | 1 |
| Hemorrhoids | 0 | 1 | 0 |
| Bloating | 0 | 2 | 0 |
| Proctitis | 0 | 0 | 2 |
| Rectal mucositis | 0 | 0 | 1 |
| Flatulence | 0 | 0 | 1 |
| Rectal pain | 0 | 0 | 1 |
| **Genito-urinary** |  |  |  |
| Nycturia | 1 | 1 | 6 |
| Pollakiuria | 0 | 1 | 7 |
| Urinary frequency | 0 | 0 | 6 |
| Urinary incontinence | 1 | 0 | 2 |
| Urinary burning/ cystitis^[[1]](#footnote-1)^ | 0 | 2 | 4 |
| Urinary discomfort | 0 | 0 | 1 |
| Urinary urgency | 0 | 0 | 2 |
| Dysuria | 0 | 2 | 7 |
| Mictalgia/ urinary tract pain | 0 | 0 | 2 |
| Urinary tract obstruction | 0 | 0 | 1 |
| **Others** |  |  |  |
| Fatigue | 1 | 0 | 2 |

**Supplementary Table 2.** Late toxicity profile (a patient may be counted more than once).

| **Late toxicity profile G1-2** | **Phase I**  **9 Gy**  **N=3** | **Phase I**  **9.5 Gy**  **N=3** | **Phase I/II**  **10 Gy**  **N=27** |
| --- | --- | --- | --- |
| **Gastrointestinal** |  |  |  |
| Diarrhea | 0 | 0 | 1 |
| Constipation | 0 | 0 | 4 |
| Fecal incontinence | 0 | 0 | 1 |
| **Genito-urinary** |  |  |  |
| Nycturia | 1 | 2 | 6 |
| Pollakiuria | 0 | 4 | 13 |
| Urinary incontinence | 0 | 2 | 3 |
| Urinary burning/ cystitis^[[2]](#footnote-2)^ | 0 | 0 | 1 |
| Urinary urgency | 0 | 1 | 5 |
| Dysuria | 0 | 1 | 4 |
| Myctalgia/ urinary tract pain | 0 | 0 | 1 |
| Mucite | 0 | 0 | 1 |
| Hematuria | 0 | 1 | 0 |
| Erectile dysfunction | 0 | 0 | 8 |
| **Others** |  |  |  |
| Fatigue | 0 | 0 | 1 |

1. Cystitis non-infectious [↑](#footnote-ref-1)
2. Cystitis non-infectious [↑](#footnote-ref-2)
